# Supplementary material for: Response of Spider and Epigaeic Beetle Assemblages to Overwinter Planting Regimes and Surrounding Landscape Compositions
Source: Insects. 2023 Dec 15;14(12):951. doi: 10.3390/insects14120951 (PMC10744018; doi:10.3390/insects14120951)
Supplement: Supplementary file 1 [file insects-14-00951-s001.zip › insects-2765226-supplementary.pdf]

Electronic supplementary materials

Response of Spider and Epigaeic Beetle Assemblages to Overwinter Planting Regimes and Surrounding Landscape Compositions

Hainan Chong [1,2], Yulin Zhu [2,3], Qian Lai [2], Song Wu [2] , Ting Jiang [2] , Dandan Zhang[1,*], and Haijun Xiao [1,2*]

1 School of Grassland Science, Beijing Forestry University, Beijing, 100083, China; chonghn15@bjfu.edu.cn.

2 Institute of Entomology, Jiangxi Agricultural University, Nanchang, 330045, China; zhuyl0821@163.com (Y.Z.); ggjopj@hotmail.com (Q.L.); w15179282987@hotmail.com (S.W.); jiang-ting0315@126.com (T.J.)

3 Institute of Biological Resources, Jiangxi Academy of Sciences, Nanchang 330096, China;

* Correspondence: zhangdandan@bjfu.edu.cn(D.Z.); hjxiao@bjfu.edu.cn (H.X.)

Table S1. Species list of spiders and carabids with diet (P = predatory; O = omnivorous), body size, ballooning or no ballooning (ballooning = 1; no ballooning = 0), hunting strategy (H = ground hunter; W = web building weaver) and numbers of individuals in 2019 and 2020.

Species Code	Order	Family	Species combined	Guild<br>P=Predators, O=Omnivory	Body Size<br>mm: base on female species	Ballooning<br>0 = No ballooning;<br>1 = Ballooning	Hunting strategy<br>H=ground hunter, W=web building weaver	2019(N)	2020(N)	Reference
SP01	Araneae	Lycosidae	*Pardosa pseudoannulata*	P	8	0	H	204	296	[1,2]
SP02	Araneae	Ctenidae	*Anahita fauna*	P	NA	NA	NA	4	3	NA
SP03	Araneae	Theridiidae	*Steatoda cavernicola*	P	6.3	0	W	1	0	[1,3]
SP04	Araneae	Lycosidae	*Pardosa.sp*	P	NA	NA	NA	377	194	NA
SP05	Araneae	Agelenidae	*Spiricoelotes zonatus*	P	6	0	W	3	0	[1,3]
SP06	Araneae	Pisauridae	*Pisauridae sp.*	P	NA	NA	NA	6	0	NA
SP07	Araneae	Nesticidae	*Nesticella mogera*	P	2.6	1	W	96	2	[1,4]
SP08	Araneae	Agelenidae	*Iwogumoa illustrata*	P	6.8	0	W	1	0	[1,5]
SP09	Araneae	Lycosidae	*Pardosa laura*	P	5.1	0	H	1	1	[1,6]
SP10	Araneae	Thomisidae	*Xysticus hedini*	P	6.06	1	H	10	0	[2,7,8]
SP11	Araneae	Linyphiidae	*Erigone atra*	P	2.4	1	W	2	0	[6,8]
SP12	Araneae	Thomisidae	*Xysticus sp.*	P	NA	NA	NA	23	0	NA
SP13	Araneae	Salticidae	*Bianor angulosus*	P	5.05	1	H	1	0	[6,9]
SP14	Araneae	Clubionidae	*Castianeira flavimaculata*	P	7.5	0	H	2	1	[1,6]
SP15	Araneae	Ctenidae	*Latouchia*	P	NA	NA	NA	1	0	NA
SP16	Araneae	Lycosidae	*Trochosa ruricoides*	P	10	0	H	46	8	[1,2]
SP17	Araneae	Linyphiidae	*Erigone prominens*	P	1.7	1	W	726	1195	[6,8]
SP18	Araneae	Oxyopidae	*Oxyopidae sp.*	P	NA	NA	NA	7	0	NA
SP19	Araneae	Lycosidae	*Pirata subpiraticus*	P	6.2	0	H	4	0	[1,6]
SP20	Araneae	Gnaphosidae	*Gnaphosa sp.*	P	NA	NA	NA	14	9	NA
SP21	Araneae	Linyphiidae	*Ummeliata insecticeps*	P	2.63	1	W	2895	7563	[6,8]
SP22	Araneae	Theridiidae	*Enoplognatha diodonta*	P	9.4	0	W	273	165	[1,10]

SP23	Araneae	Araneidae	*Singa pygmaea*	P	3.1	1	W	116	301	[3,8]
SP24	Araneae	Lycosidae	*Piratula procurva*	P	5.0	0	W	239	232	[1,2]
SP25	Araneae	Salticidae	*Irura longiochelicera*	P	5.90	1	H	1	0	[6,11]
SP26	Araneae	Gnaphosidae	*Zelotes asiaticus*	P	6.20	0	H	5	0	[1,2,6]
SP27	Araneae	Agelenidae	*Allagelena*	P	NA	NA	NA	1	0	NA
SP28	Araneae	Hahniidae	*Hahnia zhejiangensis*	P	2.90	1	W	15	3	[1,12]
SP29	Araneae	Lycosidae	*Wadicosa fidelis*	P	7.20	0	H	3	2	[1,2,13]
SP30	Araneae	Linyphiidae SP1	*Linyphiidae sp1.*	P	NA	NA	NA	9	3	NA
SP31	Araneae	Linyphiidae SP2	*Linyphiidae sp2.*	P	NA	NA	NA	14	5	NA
SP32	Araneae	Linyphiidae SP3	*Linyphiidae sp3.*	P	NA	NA	NA	22	40	NA
SP33	Araneae	Linyphiidae SP4	*Linyphiidae sp4.*	P	NA	NA	NA	11	25	NA
SP34	Araneae	Erigonidae	*Erigonidium graminicolum*	P	3.60	1	W	215	158	[6]
SP35	Coleoptera	Carabidae	*Pterostichus liodactylus*	P	8.26	——	——	56	179	[14]
SP36	Coleoptera	Carabidae	*Agonum japonicum*	P	9.61	——	——	422	235	[15]
SP37	Coleoptera	Carabidae	*Agonum chalcomus*	P	9.09	——	——	59	425	[16]
SP38	Coleoptera	Carabidae	*Harpalus sinicus*	O	10.00	——	——	4	12	[1]
SP39	Coleoptera	Carabidae	*Synuchus arcuaticollis*	P	6.25	——	——	19	0	[17]
SP40	Coleoptera	Carabidae	*Bembidion perditum*	P	5.29	——	——	53	0	[18]
SP41	Coleoptera	Carabidae	*Stenolophus castaneipennis*	P	7.37	——	——	24	40	[19]
SP42	Coleoptera	Carabidae	*Stenolophus kurosai*	P	6.70	——	——	8	0	[20]
SP43	Coleoptera	Carabidae	*Loxoncus circumcinctus*	P	10.03	——	——	4	0	[21]
SP44	Coleoptera	Carabidae	*Amara congrua*	0	9.62	——	——	4	1	[22,23]
SP45	Coleoptera	Carabidae	*Pterostichus microcephalus*	P	12.28	——	——	3	0	[24]
SP46	Coleoptera	Carabidae	*Harpalus chalcentus*	O	14.65	——	——	1	0	[24]

Table S2. Landscape cover (%) and landscape diversity (Shannon diversity index) at 1000 m radius across 13 sites.

sites		Fallow	Oilseed rape	Grassland	Forest	Ridge	SNH	Shannon diversity
	Max	0.56	0.11	0.19	0.71	0.08	0.77	0.77
13sites	Min	0.09	0.00	0.01	0.07	0.01	0.16	1.18
	Average	0.29	0.04	0.08	0.38	0.03	0.50	0.50
S01	——	0.42	0.01	0.08	0.20	0.05	0.33	1.88
S02	——	0.56	0.06	0.01	0.07	0.08	0.16	1.59
S03	——	0.33	0.11	0.19	0.18	0.05	0.41	2.19
S04	——	0.33	0.07	0.08	0.18	0.04	0.31	2.12
S05	——	0.11	0.00	0.06	0.70	0.02	0.77	1.18
S06	——	0.35	0.03	0.05	0.42	0.05	0.53	1.59
S07	——	0.28	0.04	0.08	0.40	0.01	0.49	2.09
S08	——	0.51	0.03	0.10	0.17	0.05	0.32	1.72
S09	——	0.10	0.02	0.11	0.50	0.02	0.64	1.86
S10	——	0.31	0.01	0.04	0.56	0.02	0.62	1.24
S11	——	0.32	0.01	0.15	0.28	0.04	0.48	2.12

S12	——	0.09	0.09	0.07	0.60	0.01	0.68	1.57
S13	——	0.09	0.04	0.04	0.71	0.01	0.76	1.26

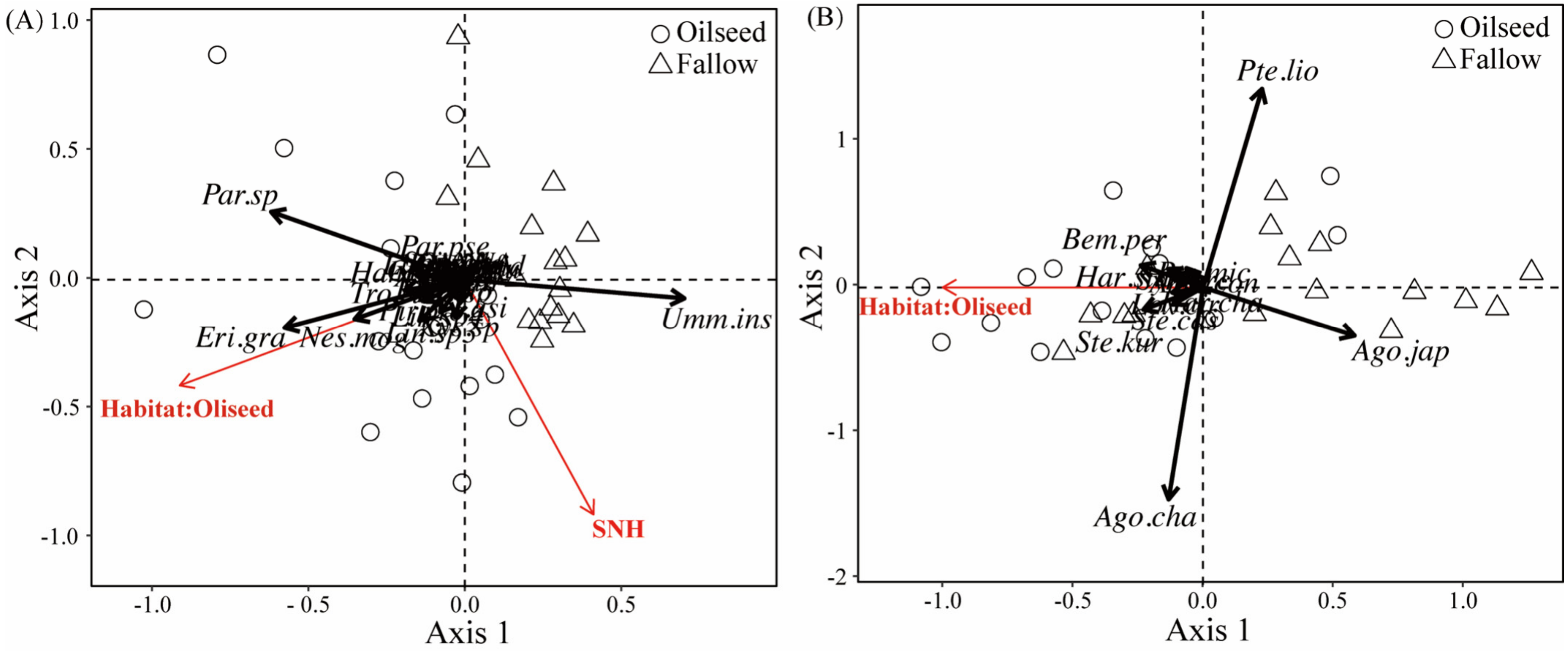

Figure S1. RDA ordination diagrams of spiders (A) and carbides (B) in the FR and the OSR, significant landscape variables and all indicator species (crosses) along the first and second RDA axe. 1, *Pardosa pse*, *Pardosa pseudoannulata*; 2, *Anahita fau*, *Anahita fauna*; 3, *Steatoda cav*, *Steatoda cavernicola*; 4, *Pardosa.sp*; 5, *Spiricoelotes zon*, *Spiricoelotes zonatus*; 6, *Pisauridae*; 7, *Nesticella mog*, *Nesticella mogera*; 8, *Iwogumoa ill*, *Iwogumoa illustrata*; 9, *Pardosa lau*, *Pardosa laura*; 10, *Xysticus hed*, *Xysticus hedini*; 11, *Erigone atr*, *Erigone atra*; 12, *Xysticus*; 13, *Bianor ang*, *Bianor angulosus*; 14, *Castianeira fla*, *Castianeira flavimaculata*; 15, *Latouchia*; 16, *Trochosa rur*, *Trochosa ruricoides*; 17, *Erigone pro*,

Erigone prominens; 18, *Oxyopidae*; 18, *Pirata sub*, *Pirata subpiraticus*; 19, *Pirata sub*, *Pirata subpiraticus*; 20, *Gnaphosa*; 21, *Ummeliata ins*, *Ummeliata insecticeps*; 22, *Enoplognatha dio*, *Enoplognatha diodonta*; 23, *Singa pyg*, *Singa pygmaea*; 24, *Piratula pro*, *Piratula procurva*; 25, *Irura lon*, *Irura longiochelicera*; 26, *Zelotes asi*, *Zelotes asiaticus*; 27, *Allagelena*; 28, *Hahnia zhe*, *Hahnia zhejiangensis*; 29, *Wadicosa fid*, *Wadicosa fidelis*; 30, *Linyphiidae sp1*; 31, *Linyphiidae sp2*; 32, *Linyphiidaesp3*; 33, *Linyphiidae sp4*; 34, *Erigonidium gra*, *Erigonidium graminicolum*; 35, *Pterostichus lio*, *Pterostichus liodactylus*; 36, *Agonum jap*, *Agonum japonicum*; 37, *Agonum cha*, *Agonum chalcomus*; 38, *Harpalus sin*, *Harpalus sinicus*; 39, *Synuchus arc*, *Synuchus arcuaticollis*; 40, *Bembidion per*, *Bembidion perditum*; 41, *Stenolophus cas*, *Stenolophus castaneipennis*; 42, *Stenolophus kur*, *Stenolophus kurosai*; 43, *Loxoncus cir*, *Loxoncus circumcinctus*; 44, *Amara con*, *Amara congrua*; 45, *Pterostichus mic*, *Pterostichus microcephalus*; 46, *Harpalus cha*, *Harpalus chalcentus*.

supplementary reference

1. Duan, M.; Liu, Y.; Li, X.; Wu, P.; Hu, W.; Zhang, F.; Shi, H.; Yu, Z.; Baudry, J. Effect of present and past landscape structures on the species richness and composition of ground beetles (Coleoptera: Carabidae) and spiders (Araneae) in a dynamic landscape. *Landscape Urban Plan*. **2019**, *192*, 103649. https://doi.org/10.1016/.2019.103649.
2. Zhang, Z.; Wang, L. *Chinese Spiders Illustrated*; Chongqing University Press: Chongqing, China, 2017; p. 924.
3. Chen, Z.; Zhang, Z. *Zoology of Zhejiang Arachnis*; Zhejiang Science and Technology Press: Hangzhou, China, 1991.
4. Kielhorn, K.H. First records of Spermophora kerinci, Nesticella mogera and Pseudanapis aloha on the European mainland (Araneae: Pholcidae, Nesticidae, Anapidae). *Arachnol. Mitt*. **2009**, *37*, 31-34. https://doi.org/10.5431/aramit3706.
5. Zhu, M.; Wang, X.; Zhang, Z. *Fauna Sinica, Invertebrata, Arachnida, Araneae, Agelenidae and Amaurobiidae*.; Science Press: Beijing, China, 2017; p. 727. https://doi.org/10.1079/ber2004350.
6. Song, D.; Zhu, M.; Chen, J. *Spiders in Agricultural Areas of China*; China Agriculture Press: Beijing, China, 1987.
7. Song, D.; Zhu, M. *Chinese Zoology: Arachnida, Arachnidea: Thomisidae, Philodromidae*; Science Press: Beijing, China, 1998.
8. Bell, J.; Bohan, D.; Shaw, E.; Weyman, G. Ballooning dispersal using silk: world fauna, phylogenies, genetics and models. *Bull. Entomol. Res*. **2005**, *95*, 69-114. https://doi.org/10.1079/ber2004350.
9. Babu, N.; Caleb, J.T.; Gopalakrishnan, P. A new distribution record of Bianor angulosus (Karsch, 1879)(Araneae: Salticidae) from Kerala, India. *J Threat Taxa*. **2021**, *13*, 19177-19180. https://doi.org/10.11609/jott.7069.13.8.19177-19180.
10. Zhu, M. *Fauna Sinica, Arachnida, Araneae, Theridiidae*; Science Press: Beijing, China, 1998.
11. Peng, X.; Yin, C. Five new species of the genus Kinhia from China (Araneae: Salticidae). *Acta Zootaxonomica Sinica.* **1991**, *16*, 35-47.

12. Song, D.; Zheng, S. Two new species of Hahnia (Araneae: Hahniidea) from ZheJiang province, China. *Sinozoologia.* **1982**, *2*, 81-84.

13. Kronestedt, T.; Zyuzin, A.A. Fixation of Lycosa fidelis O. Pickard-Cambridge, 1872 as the type species for the genus Wadicosa Zyuzin, 1985 (Araneae: Lycosiadae), with a redescription of the species. *Contributions to Natural History.* **2009**, *12*, 813-828.

14. Shi, H. *Systematic classification studies of Pterostichus in China*. Beijing Forest University, Beijing, China, 2013.

15. Smetanin, A. On the insect fauna of the Kichiga River basin, northeastern Kamchatka. *Entomol. Rev*. **2013**, *93*, 160-173, https://doi.org/10.1134/S0013873813020048.

16. Yamazaki, K.; Sugiura, S.; Kawamura, K. Ground beetles (Coleoptera: Carabidae) and other insect predators overwintering in arable and fallow fields in central Japan. *Appl. Entomol. Zool*. **2003**, *38*, 449-459. https://doi.org/10.1303/aez.2003.449.

17. Kang, B.; Lee, J.-H.; Park, J.-K. Carabid beetle species as a biological indicator for different habitat types of agricultural landscapes in Korea. J. *Ecol. Environ*. **2012**, *35*, 35-39. http://dx.doi.org/10.5141/JEFB.2012.006.

18. Chen, S. Search for common genera of Carabid in China. *Natural Enemies of Insects.* **1984**, *6*, 165-181.

19. Cai, C.; Zhu, S.; Pan, Y. *Atlas of insects from Beijing Hanshiqiao Wetland*; Science Press: Beijing, China, 2019.

20. Bates, H. Descriptions of new genera and species of Geodephayous Coleoptera, from China. *Trans. R. Entomol. Soc*. **1873**, *21*, 323-334. https://doi.org/10.1111/j.1365-2311.1873.tb00644.x.

21. Chong, H. *The effect of farmland landscape pattern on diversity and density of over-wintering spiders and carabids*. Jiangxi Agricultural University, Nanchang, 2021.

22. Sasakawa, K.; Ikeda, H.; Kubota, T. Feeding ecology of granivorous carabid larvae: a stable isotope analysis. *J. Appl. Entomol.* **2010**, *134*, 116-122. https://doi.org/10.1111/j.1439-0418.2009.01451.x.

23. Sasakawa, K. Laboratory studies on larval food habits of two syntopic, related, granivorous ground beetles Amara chalcites and A. congrua (Coleoptera: Carabidae): a

comparison with stable isotope analysis. *Appl. Entomol. Zool*. **2011**, *46*, 511-518.

24. Zhang, X.; Axmacher, J.C.; Wu, P.; Song, X.; Yu, Z.; Liu, Y. The taxon-and functional trait-dependent effects of field margin and landscape composition on predatory arthropods in wheat fields of the North China Plain. *Insect Conserv Diver.* **2020**, *13*, 328-339. https://doi.org/10.1111/icad.12403.
